# Repression of ferroptotic cell death by mitochondrial calcium signaling

**DOI:** 10.21203/rs.3.rs-3029860/v1

**Published:** 2023-07-10

**Authors:** Jianwen Chen, Bao Zhao, Shen Wang, Anjun Ma, Hong Dong, Xiang Cheng, Shengyin Lin, Xinghui Li, Laura E. Herring, Gang Xin, Qin Ma, Kai He, Ruili Xie, Yu L. Lei, Irina Ingold, Xiaolin Cheng, Zihai Li, Haitao Wen

**Affiliations:** 1 Department of Microbial Infection and Immunity, Infectious Disease Institute, The Ohio State University, Columbus, OH, USA.; 2 Pelotonia Institute for Immuno-Oncology, The Ohio State University Comprehensive Cancer Center, The Ohio State University, Columbus, OH, USA.; 3 Division of Medicinal Chemistry and Pharmacognosy, College of Pharmacy, The Ohio State University, Columbus, OH, USA.; 4 Department of Biomedical Informatics, The Ohio State University, Columbus, OH, USA.; 5 Department of Otolaryngology-Head and Neck Surgery, The Ohio State University, Columbus, OH, USA.; 6 Department of Neuroscience, The Ohio State University, Columbus, OH, USA.; 7 Proteomics Core Facility, Department of Pharmacology, University of North Carolina at Chapel Hill, Chapel Hill, NC, USA.; 8 Department of Internal Medicine, Division of Medical Oncology, The Ohio State University, Columbus, OH, USA.; 9 Department of Periodontics and Oral Medicine, University of Michigan School of Dentistry, University of Michigan Rogel Cancer Center, University of Michigan, Ann Arbor, MI 48105, USA.; 10 Department of Medicine III, Klinikum rechts der Isar, Technical University of Munich, Munich, Germany.; 11 Translational Data Analytics Institute (TDAI), The Ohio State University, Columbus, OH, USA.; 12 Present Address: Bioanalytic Laboratories, Medpace Holdings, Inc., Cincinnati, OH, USA.; 13 These authors are equally to this work.

## Abstract

The uptake of Ca^2+^ into and extrusion of calcium from the mitochondrial matrix, regulated by the mitochondrial Ca^2+^ uniporter (MCU), is a fundamental biological process that has crucial impacts on cellular metabolism, signaling, growth and survival. Herein, we report that the embryonic lethality of *Mcu*-deficient mice is fully rescued by orally supplementing ferroptosis inhibitor lipophilic antioxidant vitamin E and ubiquinol. Mechanistically, we found MCU promotes acetyl-CoA-mediated GPX4 acetylation at K90 residue, and K90R mutation impaired the GPX4 enzymatic activity, a step that is crucial for ferroptosis. Structural analysis supports the possibility that GPX4 K90R mutation alters the conformational state of the molecule, resulting in disruption of a salt bridge formation with D23, which was confirmed by mutagenesis studies. Finally, we report that deletion of MCU in cancer cells caused a marked reduction in tumor growth in multiple cancer models. In summary, our study provides a first direct link between mitochondrial calcium level and sustained GPX4 enzymatic activity to regulate ferroptosis, which consequently protects cancer cells from ferroptosis.

Mitochondrial calcium signaling is a fundamental mechanism regulating mito-metabolism by targeting key enzymes involved in the tricarboxylic acid (TCA) cycle such as PDH^1, 2^. PDH is a critical enzyme to generate acetyl-CoA, which serves as a key substrate for protein lysine acetylation (Ac-K) via the effects of a diverse set of lysine transferases^3–5^. MCU is a highly selective calcium channel essential for mito-metabolism^6–9^. This is particularly relevant to cell death regulation since pronounced rewiring of mito-metabolism usually occurs to render tumor cells resistant to therapy^10–14^. However, whether MCU-dependent mito-metabolism counteracts cell death signaling in tumor cells has not been reported.

Genetic studies have identified GPX4 as an essential repressor of ferroptosis by detoxifying peroxidized phospholipids via a tetrad catalytic motif^15–20^. Three isoforms of GPX4 have been identified (cytosolic, mitochondrial and nuclear), among which cGPX4 represents the dominant form that prevents ferroptotic cell death^17, 21^. In addition, recent studies revealed that when GPX4 enzymatic activity is inhibited, the ferroptosis suppressor protein 1 (FSP1)^22–24^ and the dihydroorotate (DHO) dehydrogenase (DHODH)^25^ protect the plasma membrane and mitochondrial inner membrane from ferroptotic damage, respectively. To date, the molecular mechanism(s) that governs GPX4 enzymatic activity has not been fully elucidated. Our preliminary results suggest MCU-mediated acetyl-CoA production is essential for GPX4 acetylation and its enzymatic activity, which correlates with its anti-ferroptotic activity. Therefore, we hypothesize that MCU promotes acetyl-CoA-mediated GPX4 acetylation, which leads to a sustained GPX4 enzymatic activity and consequently protects cancer cells from ferroptosis.

## *Mcu* deficiency induced ferroptosis caused embryonic lethality and rescued by vitamin E and ubiquinol

**Figure 1.**
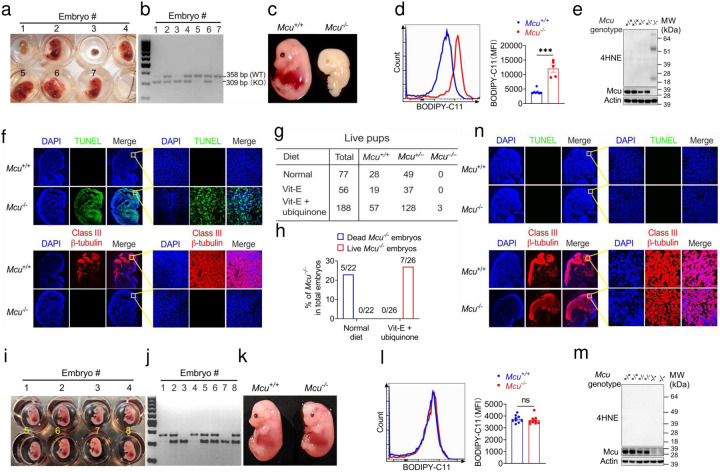
*Mcu* deficiency induced ferroptosis caused embryonic lethality and rescued by Vit-E combined with CoQ10 diet. **a-c**, Representative numbers (**a**), genotype (**b**) and morphologies (**c**) of *Mcu* wildtype and knockout mouse embryos treated with normal diet of E11.5-E13.5 embryos. **d**, Relative lipid peroxidation is indicated with BODIPY 581/591-C11 mean fluorescence intensity (MFI) of E11.5-E13.5 embryos (n≥5, *t* test). **e**, Immunoblot of 4-HNE of E11.5-E13.5 embryos. **f**, Representative immunofluorescence staining images of TUNEL and class III β-tubulin of E11.5-E13.5 embryos treated with normal diet. **g**, Number of neonates treated with normal, Vit-E or Vit-E combined with ubiquinone (CoQ10) diet, respectively. **h**, Incidence of neonates treated with normal or Vit-E combined with CoQ10 diet, respectively. **i-k**, Representative numbers (**i**), genotype (**j**) and morphologies (**k**) of *Mcu* wildtype and knockout mouse embryos treated with Vit-E combined with CoQ10 diet of E14.5 embryos. **l**, Relative lipid peroxidation of E14.5 embryos (n=9, t test). **m**, Immunoblot of 4-HNE of E14.5 embryos. **n**, Representative immunofluorescence staining images of TUNEL and class III β-tubulin of E14.5 embryos treated with Vit-E combined with CoQ10 diet. Each dotted line on the graph is representative of a single mouse. Data are representative of four independent experiments. Statistical significance was calculated with by unpaired Student’s *t*-test with *p* values noted in the figure, *p < 0.05,**p < 0.01,***p < 0.001, ns, no significant difference. The data represent mean ± SD.

We generated mice heterozygous *Mcu* mice (*Mcu*^+/−^) in C57BL/6J background and intercrossed them for homozygous, while *Mcu*^+/+^ and *Mcu*^+/−^ mice appear normal, *Mcu*^−/−^ mice die between embryonic day 11.5 (E11.5) and 13.5 (E13.5) with no live births, indicating that *Mcu* deficiency results in embryonic lethality (Fig. 1a–c), consistent with previous study^26^. We analyzed four major cell death pathways, including ferroptosis, apoptosis, pyroptosis and necroptosis, further identified induced ferroptosis in *Mcu*^−/−^ embryonic cells between E11.5 and E13.5, evidenced by increased staining with BODIPY-C11 (Fig. 1d) and elevated amounts of 4-hydroxynonenal (4-HNE)-conjugated proteins (Fig. 1e), two hallmarks of ferroptotic cell death^20, 27–29^. No apoptosis-, pyroptosis-, or necroptosis-associated markers were detected in *Mcu*^−/−^ embryos (Extended Data Fig.1a-c). We next performed TUNEL and neuron-specific class III β-tublin (Tuj1) staining assay between E11.5 and E13.5 embryos. *Mcu*^−/−^ embryos showed plenty cell death with a large number of TUNEL positive cells and severe brain degeneration with a large number of Tuj1 negative cells (Fig. 1f), a marker of neurons in the central and peripheral nervous systems from the early stage of neural differentiation. Feeding breeding mice with the diet containing vitamin E (Vit-E) and ubiquinol (a soluble analog of CoQ10), two chemicals with potent lipophilic antioxidant activity^30–32^, genotyping of new born live pups at 3 weeks revealed some viable *Mcu*^−/−^ mice, but they were obtained at a lower Mendelian ratio (Fig. 1g), suggesting that some embryos die afterwards. We next analysis status of embryos isolated at different times of gestation between E14.5 and E18.5. The Vit-E and ubiquinol diet completely rescued the embryonic lethality of *Mcu*^−/−^ on E14.5 with normal Mendelian ratio (Fig. 1h–k) and abolished BODIPY-C11 fluorescence staining and 4-HNE in *Mcu*^−/−^ cells (Fig. 1l, m). Further analysis showed that the embryonic lethality of *Mcu*^−/−^ on E16.5 were fully rescued by Vit-E and ubiquinol diet, including embryonic alive with normal Mendelian ratio (Extended Data Fig.1d-f,1p) and abolished BODIPY-C11 fluorescence staining, 4-HNE and cell death in *Mcu*^−/−^ cells (Extended Data Fig.1g-i). However, the embryonic lethality of *Mcu*^−/−^ on E18.5 were only partially rescued and massive embryos were died from ferroptosis and with a lower Mendelian ratio, a ratio of 2/21 was alive but 4/21 were dead (Extended Data Fig.1j-p). We also confirmed this phenotype by TUNEL and Tuj1 staining assay (Extended Data Fig.1o). In addition, to bypass early embryonic lethality of *Mcu*^−/−^ mice and to address whether *Mcu* deficiency is able to sustain viability and normal development in adult mice, we cross-bred *Mcu*^*f/f*^-ER-Cre mice with mice harboring loxP-flanked (floxed) *Mcu* alleles and transgenic for tamoxifen (TAM)-inducible ER-Cre. After TAM injection, we found there is no striking pathological differences between *Mcu*^*f/f*^-ER-Cre and *Mcu*^*f/f*^ group, by monitoring body weight change and performing hematoxylin-eosin staining in brain, kidney, heart and spleen (Extended Data Fig.2a-c). Collectively, these findings suggest that MCU suppresses ferroptosis activation during embryonic development. *Mcu* deficiency induced ferroptosis and caused embryonic lethality in mice.

### Acetylation of GPX4 on K90 is critical for its enzymatic activity

**Figure 2.**
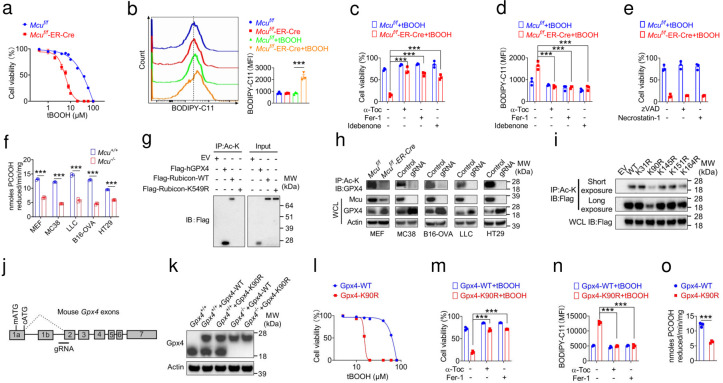
Acetylation of GPX4 on K90 is critical for its enzymatic activity. **a**, The tert-butyl hydroperoxide (tBOOH) elicited cell death in *Mcu*^*f/f*^-ER-Cre and *Mcu*^*f/f*^ MEFs, the MEFs pre-treated with 2 μM 4-hydroxytamoxifen (4-OH) for 72h. **b**, Relative lipid peroxidation as assessed by BODIPY 581/591-C11 staining treated with tBOOH in *Mcu*^*f/f*^-ER-Cre and *Mcu*^*f/f*^ MEFs. **b**, Relative lipid peroxidation as assessed by BODIPY 581/591-C11 staining treated with tBOOH in *Mcu*^*f/f*^-ER-Cre and *Mcu*^*f/f*^ MEFs. **c**, The ferroptosis inhibitors α-tocopherol (α-Toc), ferrostatin-1 (Fer-1) and idebenone prevented cell death induced by tBOOH in *Mcu*^*f/f*^-ER-Cre and *Mcu*^*f/f*^ MEFs, respectively. **d**, The ferroptosis inhibitors α-Toc, Fer-1 and idebenone prevented cell lipid peroxidation induced by tBOOH in *Mcu*^*f/f*^-ER-Cre and *Mcu*^*f/f*^ MEFs, respectively. **e**, The apoptosis inhibitor inhibitor zVAD and necrosis inhibitor necrostatin-1 didn’t rescue cell death induced by tBOOH in *Mcu*^*f/f*^-ER-Cre and *Mcu*^*f/f*^ MEFs. **f**, Substantial lower GPX4-specific enzymatic activity was detected in *Mcu* knockout cells compared to in control group using PCOOH as a substrate in MEFs, MC38, LLC, B16-OVA and HT29 cells, respectively. **g,** Immunoblot analysis of the acetylated human GPX4 in 293T, Rubicon WT and Rubicon K549R as a positive and negative control, respectively. **h**, Immunoblot analysis show that the acetylated GPX4 is reduced in *Mcu* knockout MEFs, MC38, LLC, B16-OVA and HT29 cells, respectively. **i**, Immunoblot analysis of the acetylated GPX4 on K31, K90, K145, K151, K164 in 293T, respectively. **j**, Genomic structure of the mouse *Gpx4* exons and gRNA locus. **k**, Immunoblot analysis of the GPX4 knockout MC38 cells re-overexpression Gpx4-WT or Gpx4-K90R, respectively. **l**, The tBOOH elicited cell death in *Gpx4*^−/−^-Gpx4-WT and *Gpx4*^−/−^-Gpx4-K90R MC38 cells. **m**, The ferroptosis inhibitors α-Toc and Fer-1 prevented cell lipid peroxidation induced by tBOOH in *Gpx4*^−/−^-Gpx4-WT and *Gpx4*^−/−^-Gpx4-K90R MC38 cells. **n**, The ferroptosis inhibitors α-Toc and Fer-1 prevented cell death induced by tBOOH in *Gpx4*^−/−^-Gpx4-WT and *Gpx4*^−/−^-Gpx4-K90R MC38 cells, respectively. **o**, Substantially lower GPX4-specific enzymatic activity was detected in *Gpx4*^−/−^-Gpx4-WT and *Gpx4*^−/−^-Gpx4-K90R MC38 cells compared to in control group using PCOOH as a substrate. Data are representative of four independent experiments. Statistics were assessed using two-way ANOVA, *p < 0.05,**p < 0.01,***p < 0.001, ns, no significant difference. Data represent the mean of ± SD.

To explore the molecular and biochemical mechanisms how *Mcu* regulate the cell death pathway, mouse embryonic fibroblasts (MEF) were isolated from *Mcu*^*f/f*^-ER-Cre and *Mcu*^*f/f*^ between E13.5 and E14.5 after gastrulation with the diet containing vitamin E (Vit-E) and ubiquinol, then treated with 4-hydroxytamoxifen (4-OH) on different concentrations and time points and found that 2 μM combined with 72 h is a suitable condition for 4-OH treatment (Extended Data Fig.3a). Next, we asked which cell death modality is triggered in *Mcu* deficiency cells. As illustrated, *Mcu*^*f/f*^-ER-Cre with 4-OH treatment exhibited significantly enhanced sensitivity to peroxide-induced cell death and elevated BODIPY-C11 fluorescence straining when using four ferroptosis sensitizers including tert-butyl hydroperoxide (tBOOH), arachidonic (AA), adrenic acid (ADA) and docosahexaenoic acid (DHA) (Fig. 2a–b and Extended Data Fig.3b-c). The peroxide-induced cell death could be fully rescued in *Mcu*^*f/f*^-ER-Cre cells by several ferroptosis inhibitors, including α tocopherol (α-Toc), ferrostatin-1 (Fer-1) and idebenone (a cell-permeable homolog of ubiquinone), respectively (Fig. 2c–d and Extended Data Fig.3d-e), but not by inhibitors targeting other cell death paradigms, such as apoptosis and necroptosis (Fig. 2e). These results suggest that *Mcu* sustain the cell ferroptosis in a specific manner. Moreover, similar results were confirmed by a mouse colorectal carcinoma cell line MC38 and a human colorectal cell line HT29 MCU knockout cells (Extended Data Fig.3f-o). These results suggest that *Mcu* deletion sensitizes cells to ferroptosis.

The selenoenzyme glutathione peroxidase (GPX4) is a key regulator of ferroptosis based on previous studies^20, 33^. We detected significantly decreased GPX4 enzymatic activity in several *Mcu* deficient murin and human cell lines, including *Mcu*^*f/f*^-ER-Cre, MC38, Lewis lung carcinoma (LLC), B16-OVA and HT-29 (Fig. 2f). Acetyl-CoA is an essential metabolite for protein lysine acetylation (Ac-K), both in histones and non-histone proteins^34–37^. Our recent study identified MCU as a critical mediator of acetyl-CoA production, which is an essential metabolite for protein acetylation^38^. Immunoprecipitation assay using anti-Ac-K agarose detected the acetylation of human cytosolic GPX4 overexpressed in 293T cells (Fig. 2g). Wild-type (WT) Rubicon and Rubicon with K549R mutation were included as positive and negative control, as we recently reported^38^. The mouse cytosolic GPX4 overexpressed in 293T cells was acetylated as well (Extended Data Fig.4a-b). Acetylation of GPX4 was further enhanced by the treatment with HDAC inhibitors trichostatin A (TSA) (Extended Data Fig.4c-d). Acetylated endogenous GPX4 was dramatically decreased in several *Mcu* deficient murin mouse and human cell lines (Fig. 2h). Since acetylation plays important roles in many biological processes and MCU as a critical mediator of acetyl-CoA production^38^, we investigated whether acetylation is involved in the regulation of GPX4 enzymatic activity. We performed mass spectrometry (MS) analysis and identified five potential acetylation sites on human GPX4, including K31, K90, K145, K151 and K164 (Extended Data Fig.4e and Supplementary Table S4-S5). To study the function of acetylation of these sites, we mutated each site by replacing a lysine with an arginine (R), we found that GPX4 with K90R mutation largely lost acetylation signal, it means that K90 in human GPX4 was a dominant acetylation site (Fig. 2i and Extended Data Fig.4f). K90 is an evolutionarily conserved residue both in human and mouse GPX4. We first overexpressed the 3×Flag-Gpx4-WT and 3×Flag-Gpx4-K90R in MC38 cells and then used a CRISPR/Cas9-based strategy to knockout endogenous GPX4 (Fig. 2j, k). To our surprise, we found that Gpx4 K90R significantly enhanced sensitivity to peroxide-induced by tBOOH, AA, ADA and DHA treatment (Fig. 2l and Extended Data Fig.4g). The peroxide-induced cell death and elevated BODIPY-C11 fluorescence straining could be fully rescued by ferroptosis inhibitors, α-Toc and Fer-1, respectively (Fig. 2m,n and Extended Data Fig.4h,i). GPX4 enzymatic activity was significantly decreased in Gpx4 K90R (Fig. 2o). In summary, acetylation of GPX4 on K90 is critical for its enzymatic activity and resulting in resistance to ferroptosis.

## MD simulations of GPX4 reveal a potential mechanism by which GPX4 acetylation regulates its enzymatic activity

**Figure 3.**
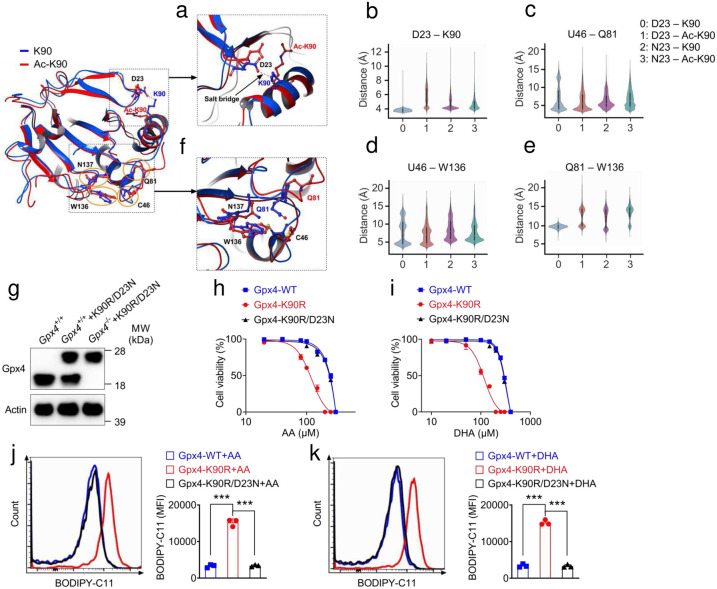
D23N mutation restored cell viability due to reduced ferroptosis. **a**, Comparison of the salt bridge structures of GPX4-K90 (Blue) and GPx4-Ac-K90 (Red) sampled in the corresponding MD simulations. **b-e**, Violin plots of distance distributions for the four simulated GPX4 systems, D23-K90 (**b**), U46-Q81 (**c**), U46-W136 (**d**), Q81-W136 (**e**). **f**, Comparison of the conformational changes of the catalytic site of GPX4-K90 (Blue) and GPX4-Ac-K90 (Red) sampled in the corresponding MD simulations. **g**, Immunoblot analysis of the GPX4 knockout MC38 cells restored by Gpx4-K90R-D23N. **h**,**i**, The AA (**h**) and DHA (**i**) elicited cell death in Gpx4-WT, *Gpx4*^−/−^-Gpx4-K90R and *Gpx4*^−/−^-Gpx4-K90R-D23N MC38 cells, respectively. **j**,**k**, Relative lipid peroxidation as assessed by BODIPY 581/591-C11 staining treated with AA (**j**) and DHA (**k**) Gpx4-WT, *Gpx4*^−/−^-Gpx4-K90R and *Gpx4*^−/−^-Gpx4-K90R-D23N MC38 cells, respectively. Data are representative of three or four independent experiments. Statistics were assessed using one-way ANOVA, *p < 0.05,**p < 0.01,***p < 0.001, ns, no significant difference. Data represent the mean of ± SD.

**Figure 4.**
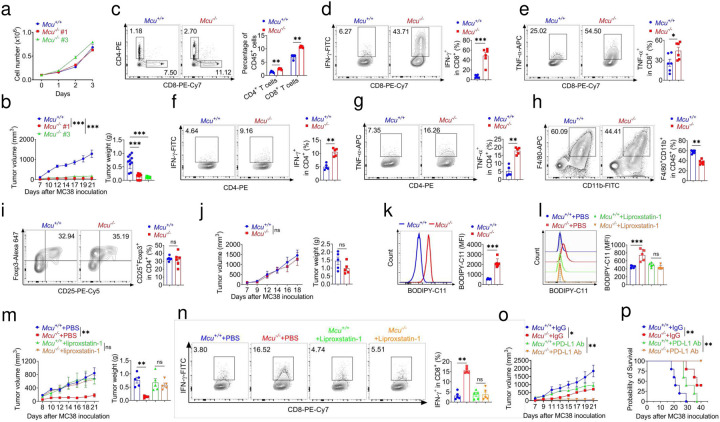
Deletion of MCU improves antitumor due to increased ferroptosis. **a**, The cell proliferation growth curve of MCU knockout MC38 cells *in vitro*. **b**, Tumor growth curve and weight of *Mcu*^+/+^ or *Mcu*^−/−^ MC38 cells (5×10^5^) in C57BL/6J mice. **c**-**i**, Flow cytometry analysis showing percentage of CD4^+^ and CD8^+^ T cells (**c**), IFN-γ^+^CD8^+^ (**d**), TNF-α^+^CD8^+^ (**e**), IFN-γ^+^CD4^+^ (**f**), TNF-α^+^CD4^+^ (**g**), F4/80^+^CD11b^+^ macrophage cells (**h)** and CD25^+^Foxp3^+^ Treg cells (**i**) of *Mcu*^+/+^ or *Mcu*^−/−^ MC38 tumors in C57BL/6J mice. **j**, Tumor growth curve and weight of *Mcu* ^+/+^ or *Mcu*^−/−^ MC38 cells (5×10^5^) in C57BL/6J mice in *Rag2*^−/−^ mice. **k**, BODIPY 581/591-C11 staining of *Mcu*^+/+^ or *Mcu*^−/−^ MC38 tumors in C57BL/6J mice. **l-n**, The ferroptosis inhibitor liproxstatin-1 prevented *Mcu*^−/−^ MC38 tumors lipid peroxidation (**l**) tumor growth curve and weight (**m**) IFN-γ^+^CD8^+^ (**n**) *in vivo*. **o**,**p**, Tumor growth curve, weight (**o**) and survival (**p**) of *Mcu*^+/+^ or *Mcu*^−/−^ MC38 cells (1×10^6^) injected with either control IgG or PD-L1 Ab at day 7, 10 and 13 post tumor inoculation in C57BL/6J mice. Data are representative of three or four independent experiments. Statistical significance was determined by unpaired Student’s t-test, two-way ANOVA, *p < 0.05,**p < 0.01,***p < 0.001, ns, no significant difference. Data represent the mean of ± SD.

To understand how GPX4 acetylation affects its enzyme activity, we first inspected the crystal structure of human GPX4 (PDB 5H5Q)^39^, and found that K90, without acetylation, forms a salt bridge with D23 (Fig. 4a). We hypothesized that acetylation of K90 would break this salt bridge and consequently lead to conformational changes of the catalytic site, affecting enzyme activity. To test this hypothesis, we performed molecular dynamics (MD) simulations to explore the conformational dynamics of human GPX4 with K90 either acetylated or non-acetylated. For comparison, we also performed simulations on the GPX4 D23N mutant with K90 either acetylated or non-acetylated as the D23N mutation is expected to break the salt bridge as well. Our simulations show that the salt bridge persists in the GPX4 WT (non-acetylated K90) throughout the simulation while no such salt bridge is formed in the other three systems during the simulations, as indicated the distance plots between residues 23 and 90 (Fig. 4b). To probe the potential structural changes of the catalytic site as a result of K90 acetylation, we performed Principal Component Analysis (PCA) on the simulation trajectories and projected all simulated catalytic tetrad structures onto the first two principal component (PC) modes (Extended Data Fig. 5a–d). The catalytic tetrad structures clearly occupy two different regions. While the non-acetylated GPX4 WT occupies only one of the regions, the other three systems where the salt bridge is absent explore both regions. In Fig. 4a, we overlay two representative structures of the catalytic tetrad from the MD simulations of GPX4 with or without K90 acetylation and found a loop at the catalytic tetrad shows the largest variance (Fig. 4c–f and Extended Data Fig.5a-d). We next evaluate the structural changes at the catalytic site as a result of the K90 acetylation by measuring the atomic distances between the following residue pairs: GLN81-TRP136 and CYS46-TRP136. These distances have been previously used to characterize the active site conformation to assess the impact of the R152H mutation on GPX4 function^39^. As shown in Fig. 4c–e, the violin plots of both distances show clear differences between the non-acetylated GPX4 WT and the other three systems. To supply a link between the breaking of the D23-K90 salt bridge and the structural changes at the catalytic site, we compare the Root Mean Square Fluctuations (RMSFs) of GPX4 with or without K90 acetylation (Extended Data Fig.5a-d). When the salt bridge is broken, residues near the acetylated K90 show larger fluctuations. This enhanced conformational dynamic appears to spread across the α-helix from the C-terminal K90 all the way to the catalytic tetrad (GLN81), where the catalytic loop shows large fluctuations, consistent with the above PCA analysis. To test this prediction, we generated exogenous overexpressing K90R/D23N double mutant in GPX4 knockout MC38 cells, in which the mutation disrupted the salt bridge and restored the GPX4 function (Fig. 3g). Strikingly, we found that K90R/D23N double mutation completely rescued the K90R cell death and abolished increased BODIPY-C11 fluorescence staining treated with AA and DHA compared with K90R mutation in *vitro* (Fig. 3h–k). Taken together, our computational data and cell death assay *in vitro* suggest that the acetylation of GPX4 K90 can potentially modulate its enzymatic activity by disrupting the formation of a salt bridge between D23 and K90, which causes structural changes of the catalytic site and eventually leads to ferroptosis and cell death.

## *Mcu* deficiency inhibits tumor progression through enhancing antitumor immunity

Evidence is accumulating that ferroptosis has been linked to tumors which provokes lipid peroxidation and immune surveillance in tumor cells, presents potentially new avenues for antitumor therapy^40–42^. To assess whether tumor cell-intrinsic MCU affected antitumor immune responses, we generated *Mcu* deficient (*Mcu*^−/−^) MC38 murine colorectal tumor cells, *Mcu*^−/−^ lewis lung carcinoma (LLC) murine lung cancer cells and *Mcu*^−/−^ B16-OVA murine melanoma cells (Extended Data Fig.3f,p,q). The cell proliferation exhibited no significant difference between *Mcu*^−/−^ and *Mcu*^+/+^ *in vitro* assay (Fig. 4a and Extended Data Fig. 6a,j). However, we found a significant delay in tumor growth as shown in tumor volume and tumor weight in C57BL/6 mice implanted with *Mcu*^−/−^ MC38 tumors (Fig 4b), *Mcu*^−/−^ LLC tumors (Extended Data Fig. 6b) and *Mcu*^−/−^ B16-OVA tumors (Extended Data Fig. 6k), respectively, compared with *Mcu*^+/+^ tumors *in vivo*. We next examined whether the enhanced antitumor by utilizing tumor microenvironment (TME). Consistently, tumors from C57BL/6 immunocompetent mice bearing *Mcu*^−/−^ MC38 tumors compared with *Mcu*^+/+^ MC38 tumors, showed higher proportion of CD8^+^ and CD4^+^ T cells, functional IFN-γ^+^CD8^+^, TNF-α^+^CD8^+^ T cells, IFN-γ^+^CD4^+^, TNF-α^+^CD4^+^ T cells and F4/80^+^CD11b^+^ macrophages, but not Treg cells (Fig. 4c–i). The similar results were also observed in mice challenged with LLC or B16-OVA cells (Extended Data Fig. 6c-g, l-p). To determine whether Mcu deficiency is dependent upon adaptive immune system, we inoculated *Mcu*^+/+^ and *Mcu*^−/−^ MC38 cells into immunodeficient *Rag2*^−/−^ mice and tracked tumor growth. The tumor growth phenotype is disappeared between *Mcu*^+/+^ and *Mcu*^−/−^ MC38 cells (Fig. 3j), indicating their association with an impaired immune response. We speculated that *Mcu*^−/−^ deletion promotes the accumulation of lipid peroxidation and activate ferroptosis, contributing to antitumor immunity. To test this possibility, BODIPY-C11 fluorescence staining was performed and analyzed by flow cytometry for tumor cells which were isolated from *Mcu*^+/+^ and *Mcu*^−/−^ MC38 tumors bearing C57BL/6 mice. The lipid peroxidation was significantly enhanced in *Mcu*^−/−^ MC38 tumors compared with *Mcu*^+/+^ tumors (Fig. 4k). The similar results were also observed in mice inoculated with LLC or B16-OVA cells (Extended Data Fig. 6i, q). To determine the impact of pharmacological inhibition of ferroptosis on *Mcu*^+/+^ and *Mcu*^−/−^ MC38 tumor growth, we i.p. injected a tolerable dose of liproxstatin-1 into implanted MC38 tumor bearing mice, liproxstatin-1 did not significantly affect the *Mcu*^+/+^ MC38 tumor development, but restored tumor development in *Mcu*^−/−^ MC38 tumors (Fig. 4m). Furthermore, liproxstatin-1 also abrogated the enhanced proportion of IFN-γ^+^CD8^+^ T cells in *Mcu*^−/−^ MC38 tumors but not *Mcu*^+/+^ tumors (Fig. 4n). In addition, the genetic epithelial Mcu deficient mouse model proved that an Mcu loss induced the ferroptosis and enhanced the antitumor immunity, supplementing lipophilic antioxidant vitamin E and ubiquinol diet fully reversed the ferroptotic antitumor phenotype (Extended Data Fig. 7a-i). Taken together, these results demonstrated that tumor MCU affects antitumor immunity via control of tumor ferroptosis *in vivo*.

Next, we investigated whether knockout *Mcu* can synergize the antitumor effect of PD-L1 blockade in immunocompetent mice. We carried out the combination treatment of *Mcu* knockout and anti-PD-L1 neutralizing antibody in MC38 tumor bearing mice. *Mcu*^−/−^ and anti-PD-L1 comparably reduced tumor growth, and their combination therapy resulted in superior tumor suppression compared with single treatment (Fig. 4o, p). Thus, *Mcu* knockout promotes tumor immunity and sensitizes therapeutic effect to checkpoint blockade.

Finally, we assessed the potential relevance of *MCU* in human antitumor immunity. We first examined the relationship between *MCU* expression and cancer patient outcome. Based on gene expression profiles of cancer patients from The Cancer Genome Atlas (TCGA) database and Kaplan-Meier survival analysis, we found that low *MCU* expression was associated with improved overall survival (OS) in patients with breast cancer, colorectal cancer, leukemia, liver cancer, ovary cancer, pancreas cancer and uveal cancer (Extended Data Fig. 8a-f). Then, we employed Tumor Immune Dysfunction and Exclusion (TIDE) algorithm to assess the relationship between *MCU* expression and clinical response to PD-1 immunotherapy. We observed that low levels of *MCU* expression were associated with melanoma and glioblastoma which increased overall survival and prolonged progression free survival (PFS) in patients having received immunotherapy PD-1 antibody (Extended Data Fig. 8h-k). These data suggest a potential involvement of *MCU* in spontaneous and ICB-induced antitumor immunity in patients with cancer and consistent with the mouse ICB treatment.

## Aceylation of K90 is critical for the enzymatic activity of GPX4 *in vivo*

**Figure 5.**
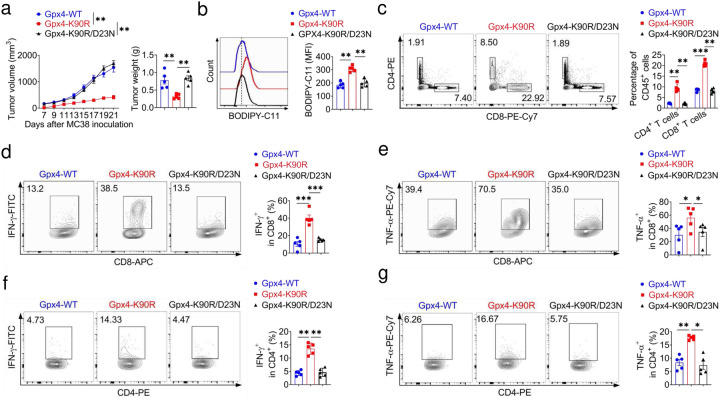
D23N mutation restored tumor growth due to reduced ferroptosis *in vivo*. **a**, Tumor growth curve and weight of Gpx4-WT, *Gpx4*^−/−^-Gpx4-K90R and *Gpx4*^−/−^-Gpx4-K90R-D23N MC38 cells (5×10^5^) in C57BL/6J mice. **b**, BODIPY 581/591-C11 staining. **c**-**g**, Flow cytometry analysis showing percentage of CD4^+^ and CD8^+^ T cells (**c**), IFN-γ^+^CD8^+^ (**d**), TNF-α^+^CD8^+^ (**e**), IFN-γ^+^CD4^+^ (**f**) and TNF-α^+^CD4^+^ (**g**) of Gpx4-WT, *Gpx4*^−/−^-Gpx4-K90R and *Gpx4*^−/−^-Gpx4-K90R-D23N MC38 tumors in C57BL/6J mice. Data are representative of three independent experiments. Statistics were assessed using one or two-way ANOVA, *p < 0.05,**p < 0.01,***p < 0.001, ns, no significant difference. Data represent the mean of ± SD.

To investigate the function of GPX4 K90 aceylation in antitumor immunity i*n vivo*, we inoculated C57BL/6 mice with *Gpx4*^−/−^ -Gpx4-WT, *Gpx4*^−/−^-Gpx4-K90R and *Gpx4*^−/−^-Gpx4-K90R/D23N MC38 cells. *Gpx4*^−/−^-Gpx4-K90R MC38 tumor bearing mice exhibited a significant delay in tumor growth as shown in volume and weight compared with *Gpx4*^−/−^-*Gpx4*-WT tumors, while *Gpx4*^−/−^-*Gpx4*-K90R/D23N MC38 tumor bearing mice showed similar tumor development with *Gpx4*^−/−^-Gpx4-WT tumors (Fig. 5a). As expected, re-expressing Gpx4-K90R in *Gpx4*^−/−^ MC38 tumors displayed significant lipid peroxidation compared with *Gpx4*^−/−^-Gpx4-WT tumors while re-expressing *Gpx4*-K90R/D23N abolished this phenotype (Fig. 5b). Furthermore, tumors from C57BL/6 mice bearing *Gpx4*^−/−^-Gpx4-K90R MC38 cells, but not *Gpx4*^−/−^-Gpx4-K90R/D23N MC38 cells, showed higher proportion of CD8^+^ and CD4^+^ T cells, functional IFN-γ^+^CD8^+^, TNF-α^+^CD8^+^ T cells, IFN-γ^+^CD4^+^, TNF-α^+^CD4^+^ T cells compared with mice bearing *Gpx4*^−/−^-Gpx4-WT tumors (Fig. 5c–g). These results implied that MCU mediated acylation of GPX4 K90 could eliminate the accumulation of lipid peroxidation and inhibit ferroptosis while knockout of *Mcu* might increase tumor ferroptosis and enhance antitumor immune response.

## Discussion

Early embryonic lethality is a common phenotype that occurs in mice that are homozygous for genetically engineered mutations. The studies mainly focus on the apoptosis and/or necroptosis pathway^43–45^, but rarely in ferroptosis pathway. In this study, we report that the embryonic lethality of *Mcu*^−/−^ mice is completely rescued by supplementing lipophilic antioxidant vitamin E (Vit-E) and ubiquinol diet. Hence, our results suggest that physiological prevention of ferroptosis is important for normal embryonic developments. *In vitro*, we demonstrate that MCU deficiency promotes ferroptosis to induce cell viability upon reduction of GPX4 enzymatic activity. Our recent study identified MCU as a critical mediator of acetyl-CoA production, which is an essential metabolite for protein acetylation^38^. The results suggest that MCU-mediated acetyl-CoA production essential for GPX4 acetylation and its enzymatic activity, which correlates with its anti-ferroptotic activity. The MS results identified K90 as a dominant acetylation site for GPX4 which is an evolutionarily conserved residue both in human and mouse. Further analysis proved K90 as a dominant site of acetylation, K90R mutation largely lost acetylation signal, impair GPX4 enzymatic activity and sensitized cells to ferroptosis *in vitro*, which is consistent with MCU deficiency. These data reveal a multipronged mechanism for the modulation of GPX4 enzymatic activity by K90 acetylation.

Recently, research efforts have been emerging on combining the power of machine deep learning and data from molecular dynamics simulations as a method of studying allosteric effects^46^. Our study suggests that K90 is a critical residue for GPX4 acetylation, we used structure-based computational modeling to study the effect of the variant on GPX4 crystal structure by bioinformatic analysis, the results suggest that GPX4 K90R is structurally different from GPX4 WT and further analysis supports the possibility that GPX4 K90R has a changed conformational state interact with D23 site by forming a salt bridge, which potentially imparis the GPX4 enzymatic activity and induce ferroptosis. These findings were confirmed by GPX4 crystal structures analysis and a series of cellular functional analysis on double mutants both i*n vitro* and *in vivo* assays. The low cost and high throughput of computational deep learning and molecular dynamics simulations would potentially benefit in driving protein structure prediction and gene targeting therapeutic strategies in a variety of diseases.

In summary, our study provides a new insight into the regulation mechanisms between *Mcu* and the ferroptosis as well as antitumor immunity. It directly links a specific metabolite, namely acetyl-CoA, to ferroptosis sensitivity and supports a growing number of studies that describe a role for cell metabolism in controlling ferroptosis^47, 48^.

## Methods

### Mice

*Mcu*^*f/f*^-ER-Cre mice and *Mcu*^*f/f*^-Villin-Cre (*Mcu*^△*IEC*^) mcie were generated by crossing the *Mcu*^*f/f*^ mice with ER-Cre mice and Villin-Cre mice, respectively. *Mcu*^+/−^ mice were generated by crossing the *Mcu*^*f/f*^ mice with EIIa-Cre mice. *Rag2*^−/−^ mice (008449), C57BL/6 mice, ER-Cre mice and Villin-Cre mice were purchased from the Jackson Laboratory. Mice between 8 to10 weeks of age were used for the animal experiments, tail genomic DNA was isolated for genotyping. Primers for genotyping PCR are listed in Supplementary Table S1. All mice were housed in specific pathogen-free facilities and all *in vivo* experiments were conducted in accordance with the National Institute of Health Guide for the Care and Use of Laboratory Animals and the Institutional Animal Care and Use Committee (IACUC). The study was approved by the Ethics Committee of The Ohio State University and all procedures were conducted in accordance with the experimental animal guidelines of The Ohio State University.

### Cell culture and stimulation

Cell lines used in this study include 293T cell line (CRL-3216), B16-OVA cell line (murine melanoma, SCC420) from Sigma-Aldrich, MC38 cell line (murine colon adenocarcinoma) was kindly provided by Dr. Yangxin Fu (UT Southwestern Medical Center), LLC cell line (murine colon cancer cell) from the American Type Culture Collection. 293T, MC38, B16-OVA and LLC cells were cultured in Dulbecco’s Modified Eagle Medium (DMEM; Gibco) supplemented with 10% fetal bovine serum (FBS; Sigma-Aldrich), 1% glutamine (Gibco), 1% sodium pyruvate, 1% non-essential amino acids (Gibco), 100 IU/ml penicillin and 100 mg/ml streptomycin (Gibco). All cell lines were maintained at 37°C, 5% CO_2_. Female animals from *Mcu*^*f/f*^-ER-Cre breeding was daily checked for vaginal mucous plug. Mouse embryonic fibroblasts (MEFs) were isolated from *Mcu*^*f/f*^-ER-Cre E13.5 to E14.5 days after gastrulation. In brief, take one embryo into a 6 cm dish with 3 ml PBS, cut off the head and remove the liver and heat abdomen, put liver into 1.5 ml tube for genotyping, move the remaining part into tube and make a cut with scissors. Centrifuge 5 min at 1500 rpm at 4 °C, remove supernatant and add 2 ml warm trypsin, vortex and incubate 20 min at 37 °C. Then add 4 ml media and 10 μg DNase I, incubate 10 min at 37 °C. Transfer the cell and add 5 ml medium to culture. The MEFs were cultured in DMEM supplemented with 10% FBS at 37°C with 5% CO_2_. The primary MEFs were isolated from our lab, 1×10^6^ cells were seeded in 10cm dish for overnight, then pre-treated with (Z)-4-hydroxy Tamoxifen (14854, Cayman) for 72 h. 2–4×10^4^ MC38, LLC, B16-OVA and HT29 knockout cells were seeded in 96 well or MC38, LLC, and HT29 pre-treated with CPI-613 (128μM, A4333, APExBIO) for overnight, then stimulated with AA (90010, Cayman), ADA (90300, Cayman), DHA (90310, Cayman), tBOOH (Sigma-Aldrich, 458139) and/or RSL3 (19288, Cayman) for 24 h. In some cases, the ferroptosis inhibitor a-Tocopherol (100 μM, T3251, Sigma-Aldrich), Ferrostatin-1 (10 μM, SML0583, Sigma-Aldrich), Idebenone (10 μM, 15475, Cayman) were simulated with ferroptosis inducer simultaneous. Cell culture supernatants were collected for cell viability by using LDH release assay. Cells were collected for cell lipid peroxidation by using BODIPY-C11 staining.

### Lipid peroxidation assay

To perform BODIPY-C11 staining, treated cells were washed and stained in 50 μl medium containing 5 μM BODIPY 581/591-C11 (D3861, Thermo Fisher Scientific), then incubated for 15 min at 37 °C in a tissue culture incubator. Cells were washed in 200 μl PBS for three times, then trypsinized and monitored immediately by 96 well flow cytometry using BD FACS Canto II (BD Bioscience). Data analysis was conducted using the FlowJo software.

### Plasmids and molecular cloning

The *Gpx4* and *MCU* gRNA were cloned into pLenti-CRISPR-V2 vector (Addgene #52961). The mouse *Gpx4* cDNA with 3’-UTR is kindly provided by Marcus Conrad. Human *Gpx4* cDNA with 3’-UTR is cloned in our lab. To generate the vector expressing *Gpx4*, mouse and human cDNA were subcloned into p3×Flag-CMV vector and pLVX-EGFP-C1 vector. To generate *Gpx4* K31R, K90R, K145R, K151R, K164R and K90R-D23N double mutation, Phusion Site-Directed mutagenesis Kit was used according to the manufacturer’s instructions (Thermo Fisher Scientific). Primers are listed are listed in Supplementary Table S2-S3, S6. Flag-Rubcion and Flag-Rubcion K549R were generate in our previous study^38^. All cloned constructs were checked by DNA sequencing.

### CRISPR/Cas9-mediated gene knockout

pLenti-CRISPR-V2 vector was used for CRISPR/Cas9-mediated gene knockout in MC38, B16-OVA, LLC and HT29 cell lines. Briefly, lentivirus vector expressing gRNA was transfected together with package vectors into 293T package cells. 48 h and 72 h after transfection, virus supernatants were harvested and filtrated with 0.2μm filter. Target cells were infected twice and 2 μg/mL puromycin was added at 3–5 days for selection. After that, the positive cells were diluted into 96-well plates at one cell per well. Isolated single clones were verified by western blot and DNA sequencing. The lentiCRISPR v2 backbone. Then, the empty vector control, *MCU*-targeted lentiviruses were packaged in 293T cells using the envelop-vector pMDL and VSV-G packaging vector. Single cell colonies were selected by unlimited dilution. Cells with effective *MCU* deletion were used for further assays. For generation gpx4 knockout MC38, first we generated the gpx4-WT/K90R/K90R-D23N overexpression MC38 and then transfected the gRNA into cells, after that the knockout cells showed as before.

### GPX4 activity assay

GPX4 activity was assessed using a GPX4-specific substrate in an enzymatically coupled test in crude whole cell lysates. Cell pellets were resuspended in 100 μl lysis buffer (100 mM KH2PO4/K2HPO4 (pH7.4), 150 mM KCl, 0.05% CHAPS, 2 mM β-mercaptoethanol, 1% protease inhibitors cocktail) followed by homogenization with 50 pestle strokes. After 15 min incubation in lysis buffer on ice, homogenate was centrifuged (4°C, 18400g, 10 min) and supernatant was transferred to a new tube. For activity measurement, 50 μl of protein supernatant from cells were added to a reaction mix consisting of 1 ml assay buffer (100 mM Tris (pH 7.8), 5 mM EDTA, 0.1% Triton X-100), 200 μM NADPH, 3 mM GSH, 0.6 U/ml glutathione reductase and 25 μM phosphatidylcholine hydroperoxide (PCOOH). GPX4 activity is determined by measuring the spectrometric decrease of 200 μM NADPH at 340 nm after being oxidized by glutathione reductase to allow the recovery of oxidized GSH which gets oxidized by GPX4 after substrate reduction. For normalization, protein concentration of the samples was measured using the Bradford method using BSA as standard according to manufacturer’s instructions. Both measurements were conducted at the SpectraMax microplate reader (Molecular Device GmbH). GPX4-specific activity was expressed as nmoles/min/mg.

### GPX4 acetylation site mapping

High resolution/accurate mass (HR/AM)-based quantitative proteomics strategy was employed to identify protein post-translational modifications. Briefly, immunoprecipitated human Flag-GPX4 or mouse GFP-Gpx4 was transfected 293T in 10cm dish respectively, after transfected 48 h, cells was lysed with RIPA buffer, Total protein extracts were incubated with Flag Agarose (1804, Sigma-Aldrich) or GFP-Trap agarose (Chromotek) overnight at 4°C under gentle agitation. Samples were washed 5 times with cold RIPA buffer and boiled with SDS buffer followed by Suspension Trapping based on-filter digestion. Three biological replicates of the pulldown experiment were included. The digests were desalted using C18 StageTips, dried in a SpeedVac and then resuspended in 20 μl LC buffer A (0.1% formic acid in water) for LC-MS/MS analysis. The analysis was performed using an Orbitrap Eclipse MS (Thermo Fisher Scientific) coupled with an Ultimate 3000 nanoLC system and a nanospray Flex ion source (Thermo Fisher Scientific). Peptides were first loaded onto a trap column (PepMap C18; 2 cm×100 μm I.D.) and then separated by an analytical column (PepMap C18, 3.0 μm; 20 cm×75mm I.D.) using a binary buffer system (buffer A, 0.1% formic acid in water; buffer B, 0.1% formic acid in acetonitrile) with a 165-min gradient (1% to 25% buffer B over 115 min; 25% to 80% buffer B over 10 min; back to 2% B in 5 min for equilibration after staying on 80% B for 15 min). MS data were acquired in a data-dependent top-12 method with a maximum injection time of 20 ms, a scan range of 350 to 1,800 kDa, and an automatic gain control target of 1e6. MS/MS was performed via higher energy collisional dissociation fragmentation with a target value of 5e5 and maximum injection time of 100 ms. Full MS and MS/MS scans were acquired by Orbitrap at resolutions of 60,000 and 17,500, respectively. Dynamic exclusion was set to 20 s. Protein identification and quantitation were performed using the MaxQuant-Andromeda software suite (version 1.6.3.4) with most of the default parameters. An UniProt mouse database (17,089 sequences) was used for the protein identification. Other parameters include trypsin as an enzyme with maximally two missed cleavage sites; protein N-terminal acetylation and methionine oxidation as variable modifications; cysteine carbamidomethylation as a fixed modification; peptide length must be at least 7 amino acids. False discovery rate was set at 1% for both proteins and peptides. MS strategy was employed to identify GPX4 acetylation sites. Briefly, immunoprecipitated GPX4 from 293T cells was subjected to SDS-PAGE. The corresponding bands were excised and the proteins were reduced with DTT, alkylated with iodoacetamide, and digested with trypsin overnight, then subjected to LC-MS/MS analysis using a Thermo Easy nLC 1200 coupled to a QExactive HF mass spectrometer. The QExactive HF was operated in data-dependent mode, and the 15 most intense precursors were selected for HCD fragmentation. Raw data files were processed using Proteome Discoverer (PD) version 2.1 (Thermo Scientific). Peak lists were searched against a reviewed Uniprot human database, appended with a common contaminants database, using Sequest. The following parameters were used to identify tryptic peptides for protein identification: 10 ppm precursor ion mass tolerance; 0.02 Da product ion mass tolerance; up to two missed trypsin cleavage sites; carbamidomethylation of Cys was set as a fixed modification; oxidation of Met, phosphorylation of Ser, Thr and Tyr, and acetylation of Lys were set as variable modifications. The ptmRS node was used to localize the sites of phosphorylation and acetylation. Peptide false discovery rates (FDR) were calculated by the Percolator node using a decoy database search and data were filtered using a 5% FDR cutoff.

### Molecular dynamics simulation of GPX4

All systems were prepared based on the crystal structure of the human GPX4 in complex with GXpep-1 (PDB 5H5Q). The structure has been slightly modified to keep the sequence identical to the human GPx4 gene, except for the Selenocysteine 46 at the catalytic site that was replaced by a cysteine residue. Previous studies have shown that the effect of this substitution on the enzyme structure and dynamics is acceptable. CHARMM GUI was used to prepare the systems. Each protein was placed in a TIP3P water box with 0.15 M KCl. The minimum distance between the protein and the box boundaries was set to 15 Å. Each solvated system was minimized, heated to 300 K, and equilibrated before the production simulation. All production simulations were performed at the NTP ensemble for a duration of 1 μs. The Monte Carlo barostat and Langevin thermostat were used to maintain the pressure at 1 atm and the temperature at 300 K, respectively. The particle Mesh Ewald (PME) was employed to calculate the long-range electrostatic interactions. All bonds involving a hydrogen atom were constrained using the SHAKE algorithm, and a time-step of 2 fs was used in all simulations. All simulations were performed with the CUDA-version Amber18 using CHARMM36 force field. For each system, 3 independent simulations were performed. Thus, a total of 12 μs trajectories were collected. Root Mean Square Deviation (RMSD) profiles showed that all systems have reached equilibrium at around 100 ns. To ensure the analysis was performed on converged simulations, we removed the first 200 ns of the trajectories, and the remaining trajectories (200 ns-1000 ns) were broken into four 200 ns intervals. Root mean square fluctuations (RMSFs) were calculated for each trajectory block, resulting in 12 RMSF values for each system, from which the corresponding mean and standard deviation were then calculated. Principal component analysis (PCA) is a dimension reduction technique that transforms a number of possibly correlated variables into a smaller number of uncorrelated variables called principal components. PCA was applied to the covariance matrices of the backbone atoms in the catalytic tetrad obtained from the MD trajectories. *k*-means algorithm with *k*=2 was used to cluster the GPX4 structures sampled from the MD simulations for visualization.

### Immunoprecipitation and immunoblotting

For immunoprecipitation assay, 293T cells were transfected with plasmids expressing empty vector, Flag-hGPX4, GFP-mGPX4 or its mutation. After 48 h, cells were lysed in RIPA buffer supplemented with complete Protease Inhibitor Cocktail (Sigma-Aldrich) for immunoprecipitation. In some cases, after transfected 40 h, cells were treated with TSA (1μM, A8183, APExBIO) 8 h. Total protein extracts were incubated with Acetyl Lysine Agarose (ICP0388–5mg, Immunechem) or GFP-Trap agarose (Proteintech) overnight at 4°C under gentle agitation. Samples were washed 5 times with cold RIPA buffer. To elute proteins from the beads, samples were incubated with 30 μl of SDS sample buffer at 95°C for 10 min. Protein content in the supernatant was analyzed by immunoblotting. For immunoblotting, electrophoresis of proteins was performed by using the NuPAGE system (Invitrogen) according to the manufacturer’s protocol. Briefly, cultured cells were collected and lysed with RIPA buffer. Proteins were separated on a NuPAGE precast gel and were transferred onto nitrocellulose membranes (Bio-Rad). Appropriate primary antibodies and HRP-conjugated secondary antibodies were used, and proteins were detected using the Enhanced Chemiluminescent (ECL) reagent (Thermo Fisher Scientific). The images were acquired with ChemiDoc MP System (Bio-Rad). Primary antibodies for immunoblotting included anti-Gpx4 (ab125066, Abcam), anti-MCU (14997, CST), anti-4HNE (MAB3249, R&D Systems), anti-GFP (sc-9996, Santa Cruz), 4HRP-conjugated anti–β-actin (sc-47778, Santa Cruz), HRP-conjugated anti-FLAG (A01869, GenScript), anti-Caspase 1 (4199T, CST),anti-Caspase 3 (14220T, CST), anti-Caspase 8 (4927S, CST), anti-p-PDH (31866S, CST), anti-RIPK1 (3493S, CST), anti-p-RIPK3 (31122S, CST), anti-RIPK3 (NBP1–77299, Novus Biologicals), anti-p-RIPK3 (81010S, CST), anti- MLKL (ab196436, Abcam), anti-MLKL (Abgent, AP14272b), anti-GSDMD was kindly provided by Vishva M. Dixit.

### Confocal microscopy

Various embryonic stages (E11.5 to E18.5) mice embryos were isolated and fixed in 4% paraformaldehyde overnight, washed 6 times each 30 minutes, put it 30% sucrose PBS overnight, put in a mold filled with Cryo-gel, freeze by dry ice and section on 20μM and immunostaining was performed according to standard procedures using anti-TUJ1 (ab52623, Abcam) and DAPI staining. TUNEL was performed on paraffin sections using the in situ cell death detection kit (Roche). Slides were deparaffinized, and sections were digested with proteinase K (20 μg/ml) for 15 min at 37°C in the presence of fluorescein-labeled dUTP. Sections were counterstained with methyl green (1 μg/ml) for 2 min. All the slides analyzed under the fluorescence microscope.

### Embryonic development analysis

*Mcu*^+/−^ mice were divided randomly into three groups fed either a vitamin E enriched, vitamin E plus every two days gavage of 2 mg CoQ10 enriched or normal diet. After mating with *Mcu*^+/−^, pregnant mice were euthanized at various embryonic development stages (E11.5 to E18.5) to obtain embryos. In brief, sterilize the chest and abdomen of the pregnant mouse with 70% ethanol. Make a ventral midline incision to open the abdomen and retrieve the uterine horn in PBS and transfer the embryo after two PBS washes to a dish. Then the embryos are used for genotyping, BODIPY581/591-C11, TUNEL, TUJ1 staining and western blot.

### Tumor models

For colitis-associated colorectal (CAC) tumor model, *Mcu*^*f/f*^ and *Mcu*^*△IEC*^ mice were divided randomly into groups fed either a vitamin E enriched, vitamin E plus CoQ10 enriched or normal diet. The induction of AOM + DSS tumorigenesis model, mice received a single intraperitoneal injection (10 mg/kg body weight) of AOM followed by three cycles of 2.5% DSS exposure for 5 d. Mice were sacrificed and tumor assessments were made 8 weeks after AOM injection. Body weight, tumor number and BIODIPY-C11 were measured for each animal at the completion of each study. For syngeneic mouse tumor models, 5×10^5^ MC38, LLC and B16-OVA *Mcu*-knockout or control cells were inoculated subcutaneously in the right flank at C57BL/6 mice or *Rag2*^−/−^ mice. For PD-L1 blockade, mice were intraperitoneally injected 1×10^6^ MC38 *Mcu*-knockout or control cells with 250 μg of control IgG or anti-PD-L1 antibody (clone 10F.9G2, Bio X Cell) at day 7, 10 and 13 post tumor cell inoculation. For rescue experiment, liproxstatin-1 (30 mg/kg) liproxstatin-1 (30 mg/kg) (B8221, ApexBio) was administered intraperitoneally every day from 7 to 20 days after post MC38 tumor cell inoculation, vitamin E plus CoQ10 diet fed the mice throughout the life cycle for B16-OVA tumor cell inoculation. For *Gpx4* rescue experiment, *Gpx4* K90R mutation and MC38 control cells were inoculated subcutaneously in the right flank at C57BL/6 mice. Mice were sacrificed at 18 days for flow cytometry and sacrificed when tumors reached a size of 2000 mm^3^ for survival curve.

### TAM induced animal model

*Mcu*^*f/f*^ and *Mcu*^*f/f*^-ER-Cre mice were divided randomly into two groups, 4-OH-tamoxifen (TAM) 2 mg/mouse in corn oil injections were performed via intraperitoneal (IP) injection for a total seven consecutive days. The body weight was monitored every two days for two weeks. After that, the mice were sacrificed and the tissues were collected for western blot and H&E staining.

### Flow cytometry

Mice tumors were dissected and weighed, then minced into small fragments and digested with 1 mg/mL collagenase IV and 50 U/mL DNase I for 30 min at 37°C. The cell suspensions were mechanically disaggregated and filtered with 100μm cell strainers. Centrifuge and lysed with the ammonium-chloride-potassium (ACK) lysis buffer for 5 min, then added PBS and passed through 100μm cell strainers. Single cell suspensions were treated with purified anti-CD16/32 (Fc receptor block, clone 93; BioLegend), and then stained with fluorochrome-conjugated monoclonal antibodies, including anti-CD11b-APC (M1/70), anti-F4/80-FITC (BM8), anti-CD4-PE (GK1.5), anti-CD8-APC (53–6.7), anti-CD8-PE-Cy7 (53–6.7), anti-CD25-PE-Cy5 (PC61) from BioLegend. For intracellular cytokine staining of tumor-infiltrating lymphocytes (TILs), cells were stimulated *in vitro* with phorbol-12-myristate 13-acetate (PMA) (50 ng/ml, Sigma-Aldrich) and ionomycin (500 ng/ml, Sigma-Aldrich) in the presence of GolgiPlug and GolgiStop (BD Biosciences) for 4 h, and then surface stained as aforementioned. Cells were then fixed and permeabilized using BD Cytofix/Cytoperm (BD Biosciences) and stained with anti-IFN-γ-FITC (XMG1.2) and anti-TNF-α-APC (MP6-XT22) from BioLegend. For intranuclear Foxp3 staining, single-cell suspensions were stained with antibodies against cell-surface antigens as aforementioned, fixed and permeabilized using Foxp3 Fix/Perm Buffer Kit (BioLegend) followed by staining with Foxp3 (clone MF-14; BioLegend).

### Statistics analysis

Statistical analysis was carried out with Prism 8.4.0 for Macintosh. All data are shown as mean ± SD. The mean values for biochemical data from each group were compared by two-tailed Student’s *t*-test. Comparisons between multiple time points were analyzed by repeated-measurements analysis of variance (ANOVA) with Bonferroni post-tests. In all tests, *P* values of less than 0.05 were considered statistically significant.
